# Biomechanical analysis of patient specific cone vs conventional stem in revision total knee arthroplasty

**DOI:** 10.1186/s13018-024-04936-0

**Published:** 2024-07-27

**Authors:** Gianluca Piovan, Edoardo Bori, Marika Padalino, Silvia Pianigiani, Bernardo Innocenti

**Affiliations:** 1 Department of Orthopaedic and Traumatology, S. Cuore-Don Calabria Hospital, Negrar, Italy; 2 BEAMS Department (Bio Electro and Mechanical Systems), École Polytechnique de Bruxelles, Université Libre de Bruxelles, Av. F. Roosevelt, 50 CP165/56, Brussels, 1050 Belgium; 3 Département ECAM, Haute Ecole ICHEC-ECAM-ISFSC, Woluwe-Saint-Lambert, Belgium; 4 Adler Ortho, Cormano, Milan, 20032 Italy

## Abstract

**Background:**

In revision total knee arthroplasty, addressing significant bone loss often involves the use of cemented or press-fit stems to ensure implant stability and long-term fixation. A possible alternative to stem was recently introduced utilizing custom-made porous metaphyseal cones, designed to reconstruct the missing tibial and femoral geometries. Early clinical and radiological assessments have shown promising results. The objective of this research was to biomechanically evaluate the performances of these custom-made cones.

**Methods:**

The biomechanical study was conducted using a validated finite element model. The bone geometries of a patient (selected for their history of four knee revisions due to infection and periprosthetic fractures, followed by a successful treatment with custom-made 3D-printed metaphyseal cones) were employed for the study. On these bone models, different revision scenarios were simulated and examined biomechanically: (A) custom-made cementless metaphyseal cones; (B) cemented stems; (C) press-fit stems; (D) distal femoral reconstruction with press-fit stem. All the models were analyzed at 0 °and 90 °of flexion, under physiological load conditions simulating daily activities; stress distribution, average Von-Mises stresses and risk of fracture were then analyzed and compared among configurations.

**Results:**

The use of custom-made 3D-printed cones exhibited the most favorable stress distribution in both femoral and tibial bones. Tibial bone stress was evenly distributed in custom-made cone configurations, while stress concentration was observed in distal regions for the other scenarios. Additionally, custom-made cones displayed overall homogeneity and lower stress levels, potentially contributing to limit pain. Symmetrical stress distribution was observed between the lateral and medial proximal tibia in custom-made cone models, whereas other scenarios exhibited uneven stress, particularly in the anterior tibial bone.

**Conclusions:**

The biomechanical analysis of porous custom-made metaphyseal cones in re-revision arthroplasties is in agreement with the positive clinical and radiological outcomes. These findings provide valuable insights into the potential benefits of using custom-made cones, which offer more uniform stress distribution and may contribute to improve patient outcomes in revision TKA procedures. Further studies in this direction are warranted to validate these biomechanical findings.

## Background

Primary Total Knee Arthroplasty (TKA) is a successful orthopedic procedure used to restore normal knee function in patients suffering from a wide group of pathological conditions [1–7]. However, pain, dissatisfaction, and implant failure can occur both in the immediate follow-up and several years after surgery [1, 4, 6, 8–15].

Failures of primary TKAs require to replace the implant, usually with a revision traditional (RT) implant [1, 12–16]. RT implants available on the market are designed with different shapes, and offer several levels of constraint in order to optimally fit the bone and soft-tissues status after the primary TKA is removed [17–22].

In contrast to primary TKA, performing revision TKA (R-TKA) presents significantly greater surgical challenges [1, 4–7, 16]: more advanced techniques are indeed required to achieve a secure and durable implant fixation, to establish a well-balanced knee joint (with equal spaces in both extension and flexion), and to manage properly bone loss and/or weak bones in extremely selected cases [14, 15].

Patients who have undergone multiple revisions (factor that usually involves negative consequences, such as significant bone loss, lack of prosthetic stability and overall extremely weakened bones), patients with severe post-implant or post-traumatic infections, or even oncological cases, can be extremely challenging in terms of achieving of proper fixation and adequate prosthetic stability, thus more invasive solutions may be necessary [23–29].

Since being able to cover for all possible scenarios with the standard “off-the-shelf” implants is challenging for the orthopedic companies, several patient-specific solutions have been introduced in the recent years.

In order to enhance this field, the introduction of powder manufacturing technology for metals processing has been embraced also for the biomedical field [30–34]. This technology has led to the development of porous implants with controlled porosity, enabling a more precise control on the mechanical properties of the implant to meet the requirements of anatomy matching, bone osteointegration, and stability in the implant region [30–33].

As an additional advantage of this manufacturing approach, the ability to specifically design the shape of the implant to match the patient’s morphology, involving in some cases very complex geometries, sets it apart from the limitations of traditional production. Furthermore, it enables the design of integrated porous regions [30, 33, 34].

Designing and producing patient-specific implants is time consuming. Unlike standard designs, these implants are not immediately available for surgery. However, the inconvenience of waiting for production is often outweighed by the need for a more long-lasting and efficient implant. It is worth mentioning that the latest additive manufacturing technologies are making significant improvements in this area, becoming increasingly faster [30, 31, 34].

Therefore, among the various solutions for treating bone loss enabled by this new technology, porous custom-made cones have emerged as one of the most clinically successful strategies adopted in recent years [14, 15, 35–41].

Nevertheless, the current literature still lacks a comprehensive understanding of the biomechanical effects and advantages of the use of custom-made implants incomparison to other possible surgical approaches.

The aim of the present research study is dual: firstly, to verify and understand the benefits of the use of custom-made cones compared to traditional revision techniques, and secondly, to analyze whether using a higher level of porosity in these devices, as opposed to conventional ones, could offer significant advantages.

This research study is performed following a biomechanical approach, by means of finite element modeling (FEM) and analysis (FEA). The FEM model is based upon a real clinical case of a patient affected by severe pathological conditions, after a RT surgery performed to assess them.

The novelty of this study lies into the investigation of several surgical solutions used during severe knee revisions, both to confirm the importance of personalized techniques and to explore scenarios related to different levels of porosity for metal orthopedic implants.

Once verified the obtained results, this study can be used to assist the clinicians in the decision-making process of knee revision surgeries approaches, especially when dealing with patients with severe bone conditions or previous complications.

## Methods

The finite element model developed in this study was based on a previously validated and published finite element model of the knee [18, 42], and it includes the features described in the following sections.

### Patient geometries

**Fig. 1:**
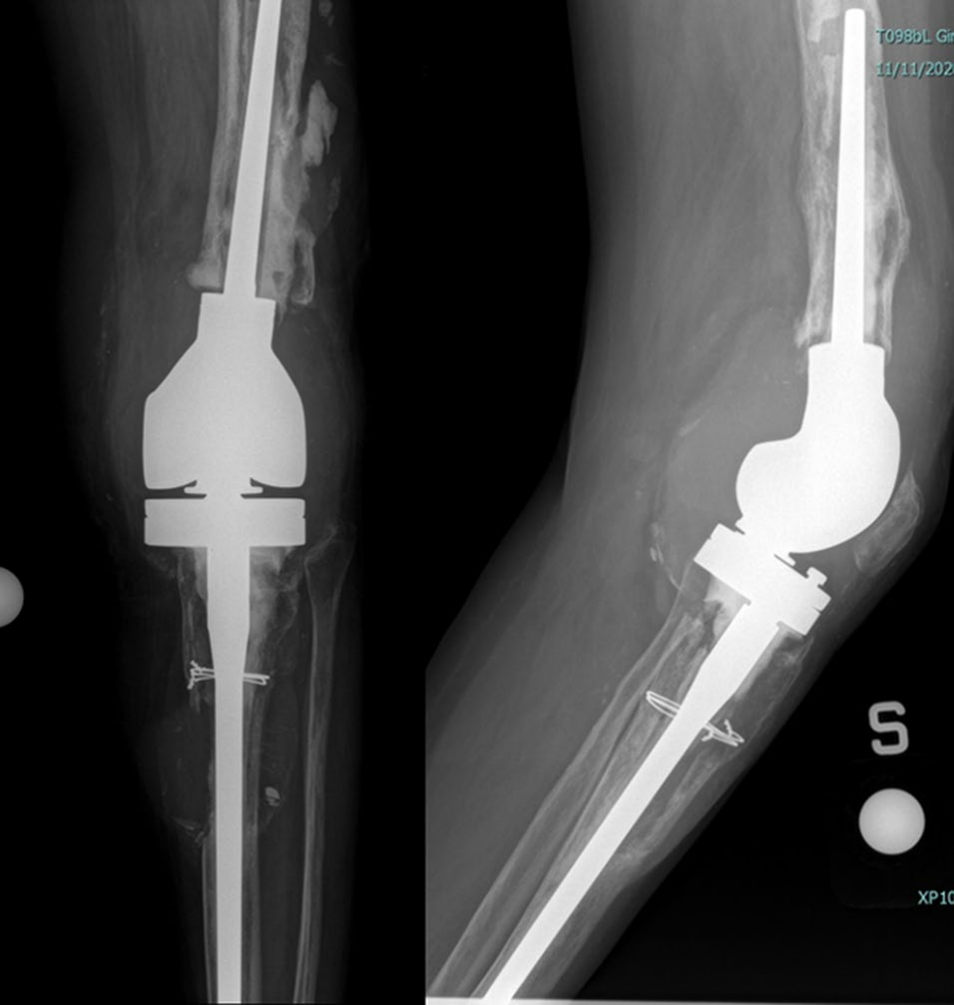
Preoperative X-rays: Aseptic loosening with severe osteolysis and pseudotumor formations around tibial component

**Fig. 2:**
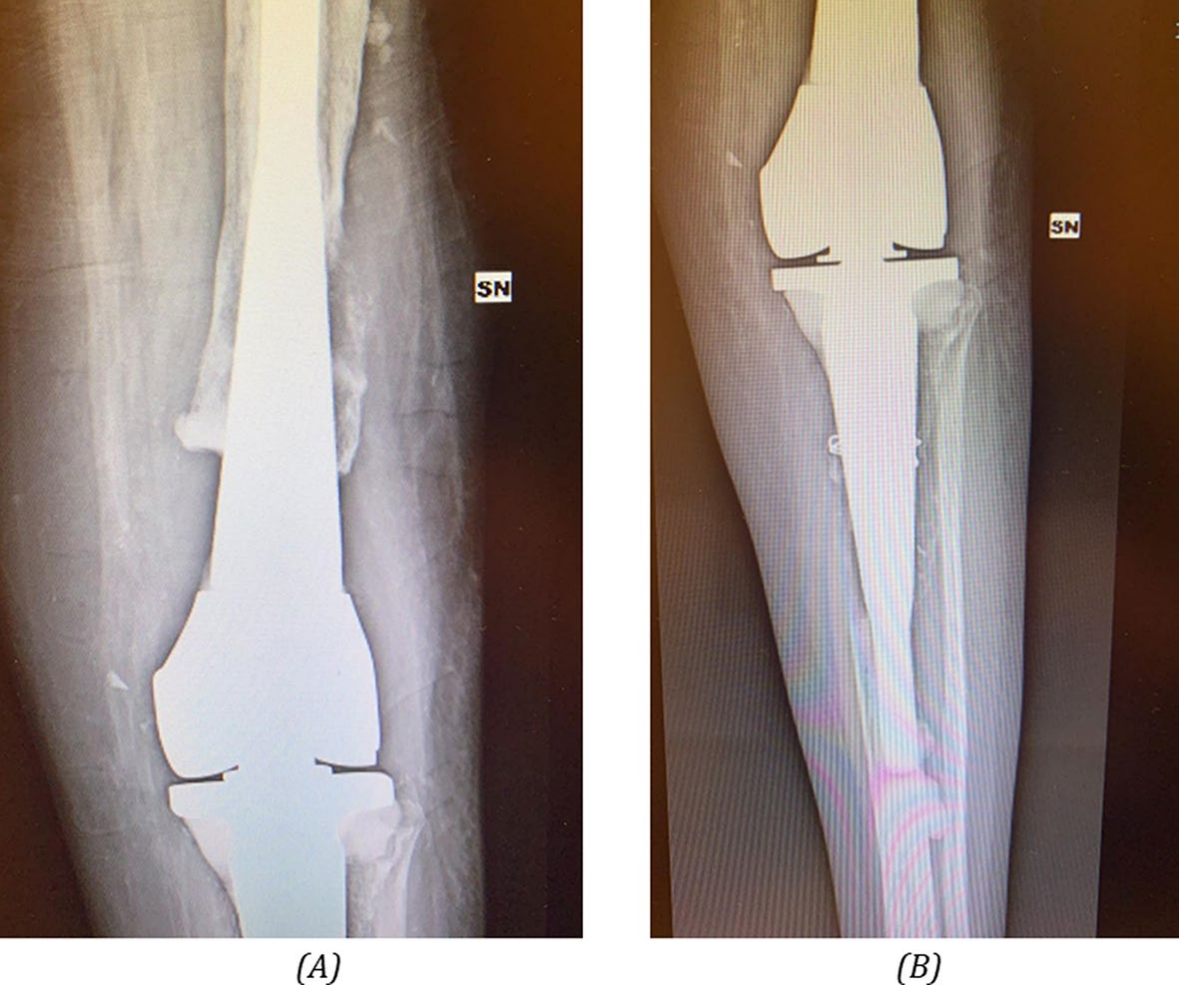
Post-implant X-rays of femoral (**A**) and tibial (**B**) custom-made cone

The patient analyzed for the study is a real surgical case who was selected to receive a RT implant: at the time of surgery, the patient was a 68 years old woman. She underwent nine previous surgical interventions on the left knee, including a medial uni-compartmental knee replacement, revision surgery with a total knee for instability, two steps revision for infection, and a further revision with a rotating hinge prosthesis for aseptic loosening. This last surgery started presenting issues at 5 years after the implant, with the onset of pain and motor disability. The diagnosis of aseptic loosening was thus performed by a multidisciplinary medical team, according to the hospital protocol for painful arthroplasty. Figures 1 and 2 shows, respectively, the preoperative and post-operative X-rays images of the analyzed patient.

**Fig. 3:**
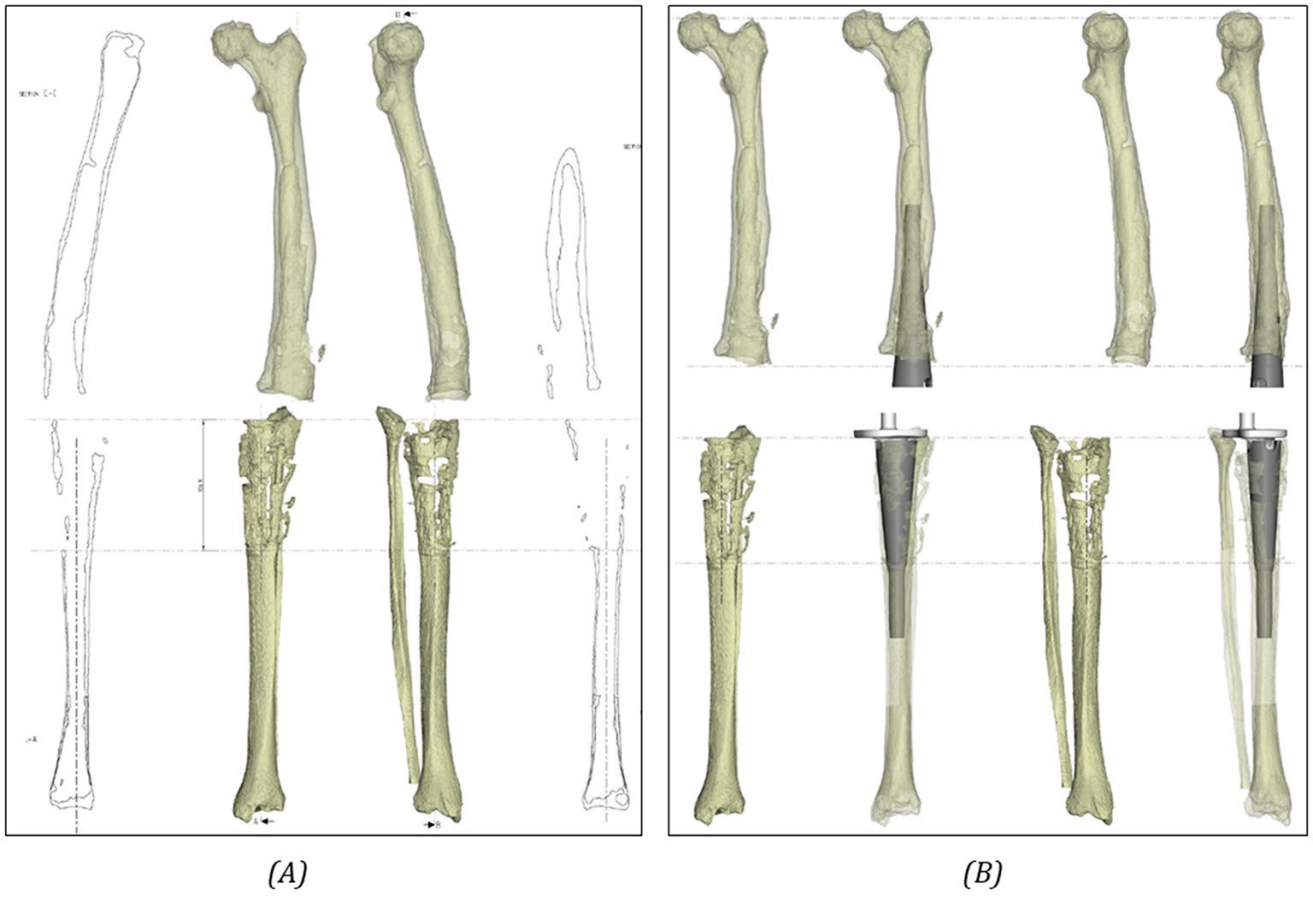
Imaging analysis: **A**) CT-based 3D reconstruction for bone loss evaluation; **B**) 3D rendering of tibial and femoral custom cones

The surgery was conducted in two stages, and the computed tomography (CT) scans were performed after implant removal to avoid issues with metal artifacts; these CT scans were then used to obtain the three-dimensional models of the patient’s femoral and tibial bones. Due to the multiple revision surgeries undergone by the patient, both the remaining patient’s femoral and tibial bone includes exclusively cortical bone. The patella was not geometrically considered for this study. Figure 3A illustrate the CT-based 3D reconstruction for bone loss evaluation.

### Implant

Two patient-specific cones, one for the femur and one for the tibia, were designed using as reference the bone geometries obtained from the medical CT scans (Fig. 3) and were optimized by the industrial manufacturer (Adler Ortho, Cormano, Italy), to properly assess the defects and precisely fit the prosthetic implants to the tibial and femoral cortical bone (following therefore a patient specific approach). In detail, the CAD design of the cone is achieved by defining the section of the implant on different axial planes, aiming to achieve the most optimal bone contact possible. Subsequently, a surface encompassing simultaneously all previously defined sections is generated. Finally, the section thickness is determined, and this parameter is allowed to vary along the length of the device. In terms of interfaces, the external surface of the cone is designed for press-fit (and thus in direct contact with the cortical bone), while the inner part is designed to host a cemented implant and it is therefore optimized for cement interaction. In order to ensure sufficient fixation of the prosthetic components, bone cement was furthermore used to close the eventual gaps between cortical bone and implants in the proximal tibial region and in the distal femoral one.

### Model implementation

The geometries of the bones and of the tibial and femoral custom-made cones were then imported in the simulation environment and positioned as they were surgically implanted in the patient’s left leg, using the medical images of the patient obtained after surgery as a reference.

Via numerical models, the use of this personalized revision technique was compared with the use of traditional revision techniques (such as the use of cemented stems, press-fit stems and a large resection prosthesis) applied on the same patient’s bones, in order to enhance the comparative significance of the output. Additionally, the differences in the application of two different material behaviors were investigated.

**Fig. 4:**
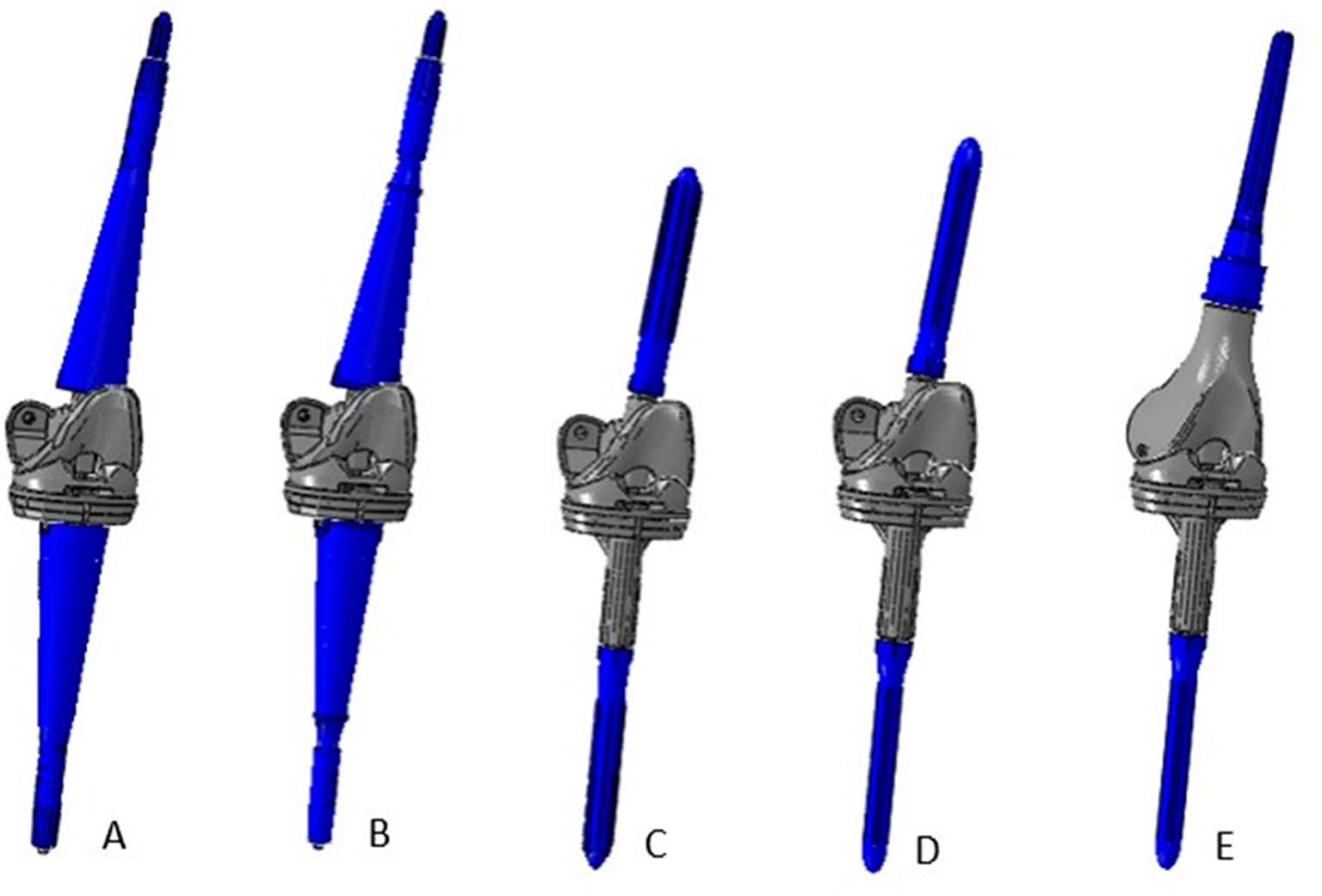
Overview of the different configurations analyzed: **A**) use of porous titanium custom-made cones, **B**) use of Ti-Por® porous titanium custom-made cones, **C**) use of press-fit stems, **D**) use of cemented stems, **E**) use of large resection prostheses coupled with an extensor element and a press-fit stem

Overall, the following configurations were analyzed (Fig. 4):

- Technique A: Revision TKA with the use of custom-made cones; the stems are thinner than the diaphyseal canals, the cones are meta-diaphyseal and are made with conventional porous metal.
- Techniques B: Revision TKA with the use of custom-made cones; the stems are thinner than the diaphyseal canals, the cones are meta-diaphyseal and made with high-level porous metal (Ti-Por^®^ Adler Ortho).
- Techniques C: Revision TKA with the use of standard press-fit stems.
- Techniques D: Revision TKA with the use of standard cemented stems.
- Techniques E: Revision TKA with the use of large resection prostheses, with press-fit stems connected to the metal prosthesis.

All considered surgical procedures were combined with a hinged total knee prosthesis (GENUS PANTHEON, Adler Ortho (Cormano, Milan, Italy)) with the exception of the technique E, involving a large resection implant (PANTHEON DRF) for what concerns the femoral component. The CAD files of the custom-made cones and prosthesis components were provided by the industrial manufacturer.

The materials properties were defined and assigned to the corresponding components according to the literature data [1, 15, 17, 29, 37, 43–45].

The main properties used to model all the materials are summarized in Table 1.

**Table 1:** Material properties and models used for the study

Material	Material Model	Young’s Modulus [MPa]	Poisson’s ratio	Mass Density [g/cm^3^]
Cortical bone	Transversely Isotropic	E1=11,500 E2=11,500 E3=17,000	V12=0.58 V13=0.31 V23=0.31	1.85
UHMWPE	Elastic isotropic	685	0.40	0.97
CoCrMo ISO 5832	Elastic isotropic	210,000	0.29	10.00
Ti6Al-4 V ISO 5832-3	Elastic isotropic	110,000	0.35	4.90
Conventional porous titanium	Elastic isotropic	25,000	0.35	4.90
PMMA	Elastic isotropic	3,000	0.35	1.30
Ti-Por^®^	Elastic isotropic	4,865	0,3	2.06*

For the cortical bone, the direction E3 represents the axial direction. * Ti-Por^®^ has a density of 42% compared to conventional porous titanium

According to the literature [15, 17, 18, 29, 37, 43, 44], linear elastic models were chosen for all the materials involved in this research study, with exception of the cortical bone that was modeled as transversely isotropic, with the principal axis corresponding to the mechanical axis of the bone. The material adopted for the cement (poly-methyl methacrylate, PMMA) was considered homogeneous and isotropic [29, 37].

The cones were always designed with the same degree of porosity (700 μm average pore size and 65% porosity). However, when considering the total cone density, it should be noted that most of the time cones have a solid wall with an ingrowth surface on top of it, featuring the parameters described above. To analyze the effect of high-porosity metal, the custom-made cones utilized in techniques A and B share the same design, but different material properties were applied for the simulation. The mechanical properties, in terms of Young’s modulus and Poisson coefficient, of conventional porous titanium were used for technique A [15]; meanwhile technique B utilized high-level porosity titanium, characterized with values modified to match with the Ti-Por^®^ overall behavior [37].

In detail, the custom-made meta-diaphyseal cones involved in the technique A are made entirely of conventional porous titanium [15]; on the opposite, the custom-made meta-diaphyseal cones used in the technique B are bi-material: titanium alloy (Ti-6Al-4 V) was used for the main structure and the different porosity was involved only for the bone interface region, thus made of Ti-Por^®^ [37].

For technique E, the geometries of the femoral component and the femoral stem are different from the other ones, as a large resection prosthesis extension element is involved (Fig. 4). In this case, an extension element is used to customize the length of the replacement, and it is applied between the stem and the femoral component. The extension element is made of titanium alloy (Ti-6Al-4 V).

For all five techniques, the material of the femoral components was a Chromium Cobalt Molybdenum alloy (CoCrMo ISO 5832) [15], the tibial inserts were made of ultra-high molecular weight polyethylene (UHMWPE) [29, 37], and the tibial components were made of titanium aluminum vanadium alloy (Ti-6Al-4 V ISO 5832-3) [29, 37].

All femoral and tibial stems involved were made of titanium aluminum vanadium alloy (Ti-6Al-4 V ISO 5832-3) [29, 37].

**Fig. 5:**
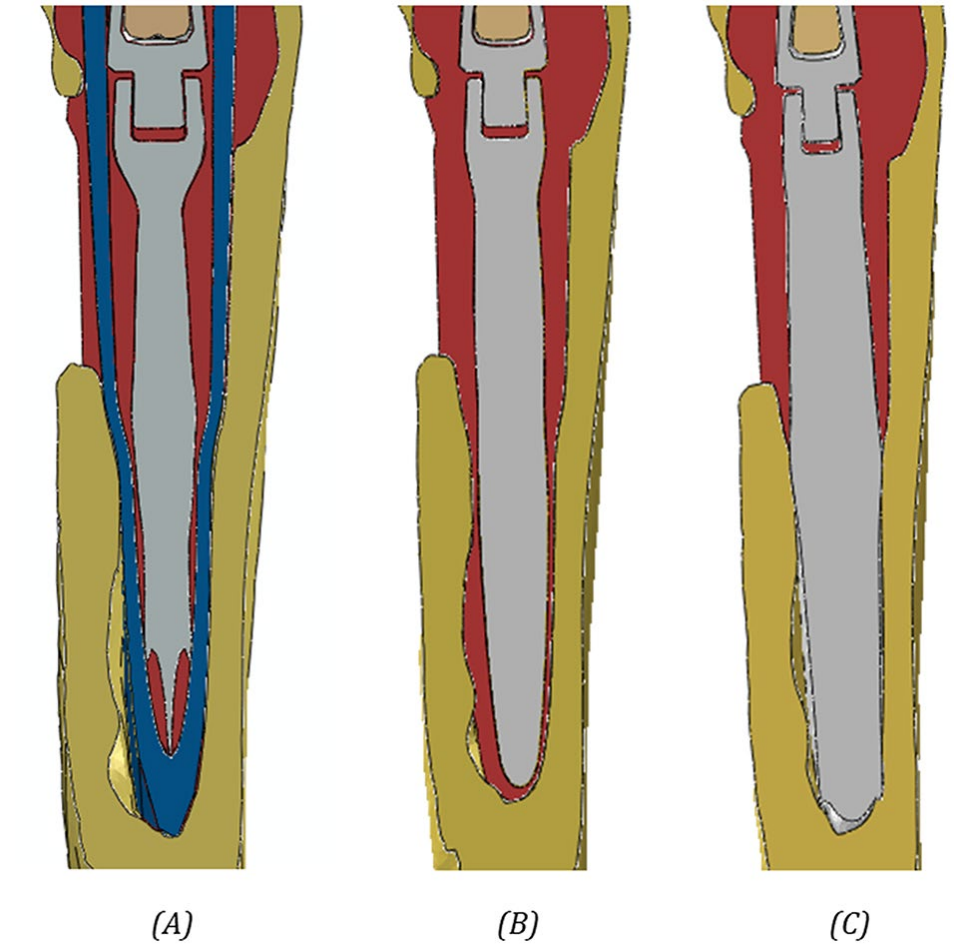
Lateral section of the distal tibial bone region, where the interaction between the cone/stem tip and the bone occurs, with the different prosthetic components analyzed. The different structures are represented by different colors: bone in yellow, cement in red, cone in blue and implant in grey. **A**) Custom cone (outer interface: press-fit; inner interface: cemented). **B**) Cemented stem. **C**) Press-fit stem. Cement is always present in the proximal region of the tibia in order to fill the gap due to the bone loss, and the differences between the cemented and press-fit approaches can be found in the respective distal regions

To highlight the substantial design differences of the five techniques, Fig. 5 represents the tibial bone with a section cut: (A) is representative of techniques A and B using custom-made cones, (B) is representative of techniques D and E using cemented stems, (C) represents technique C with press-fit stem.

**Fig. 6:**
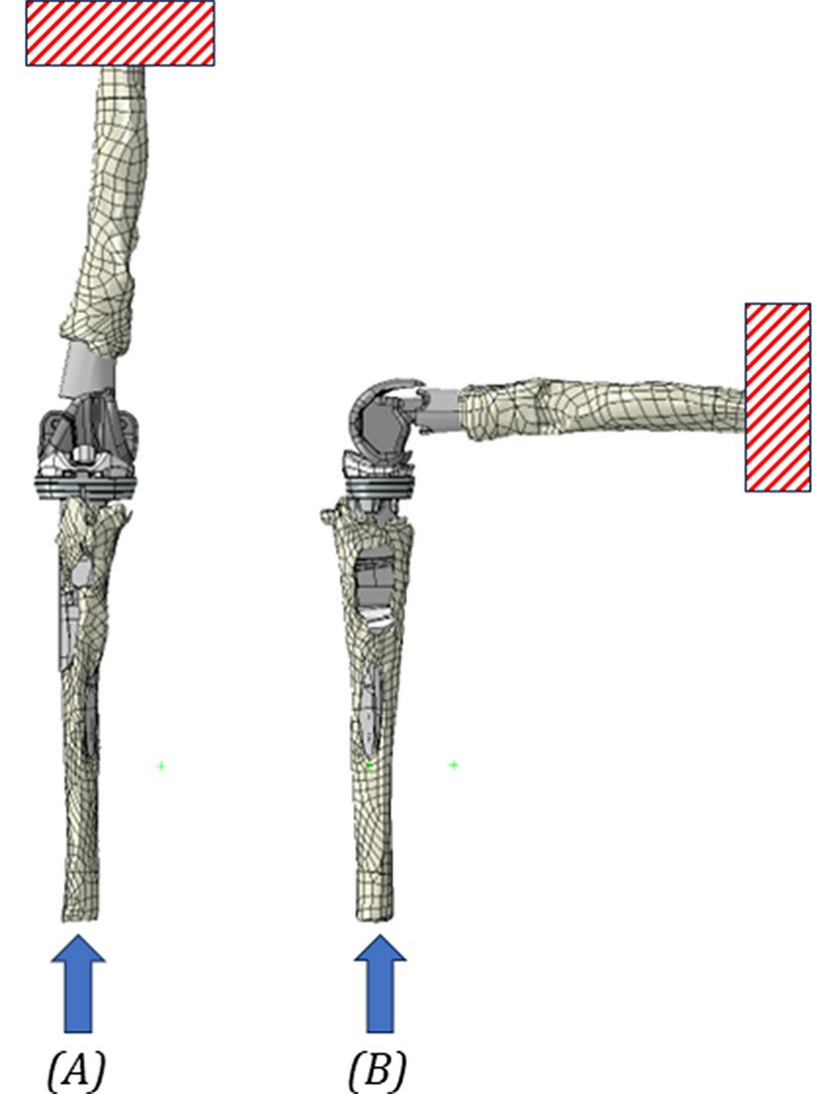
Forces and constraints applied for the two analyzed configurations: full-extension (**A**), chair-rise (**B**). The arrow represents the location and the direction of the force applied, while the dashed red rectangle indicates the region considered to be fixed

A static analysis was chosen, and the most demanding boundary conditions related to common daily activities typical of the addressed patient’s population were reproduced. In particular, for all the implant models, the following two daily activities were then implemented (Fig. 6):

- Full-extension (0° of flexion): reproduces the upright position. An axial static load (cranio-caudal direction) of 2200 N is applied on the distal surface of the tibia while the proximal femur is considered fixed. The magnitude of the force corresponds to around 3.1 times of 70 kg body weight [15, 18,29,42,44,46,].
- Chair-rise (90° of flexion): investigates the action of getting up from a sitting position. In this case the femur is fixed proximally, and a static load of 1000 N is applied on the distal surface of the tibia along its mechanical axis. The force magnitude considered is lower in comparison to other configurations, as a consequence of the patients’ common practice of using their hands for additional support when transitioning from a seated position to a standing one.

All applied static loads were in line with a published studies in the literature [9, 15, 17–20, 46–48].

All contact pairs are considered fully bonded, except with the exception of friction between the femoral component and tibial insert (µ = 0.04), the custom-made cones and tibial and femoral component, the cement and stem (µ = 0.2), in agreement with previous studies on biomechanical static analysis [15, 44, 49].

Even if the patella was not geometrically considered, its contribution is included in the boundary conditions [15, 17, 19, 43].

All the models were analyzed using Abaqus/Explicit version 2019 (Dassault Systèmes, Vélizy-Villacoublay, France), a linear tetrahedral mesh was used to mesh each structure of the models; the size of the mesh elements varied between 1 and 10, depending on the geometry. The correct meshing quality was ensured through convergence analysis [50].

For every configuration and technique examined, the average Von Mises stress was computed on different Regions of Interest (ROI) in the femoral and tibial bone. In detail, two ROIs were defined: the distal meta-diaphyseal zone of the cortical femur and the proximal meta-diaphyseal zone of the cortical tibia. Overall stress distribution patterns were assessed and the bone risk of fracture (RF) was calculated to evaluate tibial and femoral resistance to mechanical loading. RF was defined as the ratio between the maximum principal strain in the femoral or in the tibial bone (either compressive or tensile, ɛ_max_) and the corresponding ultimate strain limit (ɛ_lim_):

RF = ɛ_max ∕_ɛ_lim_.

According to the literature [15, 51, 52], the ultimate compressive strain for bone is 0.0104, while the ultimate tensile strain limit was taken as 70% of the previous value, 0.0073.

## Results

As a general statement, techniques involving the use of custom-made cones (A and B) led to similar results if compared to the other examined scenarios.

**Fig. 7:**
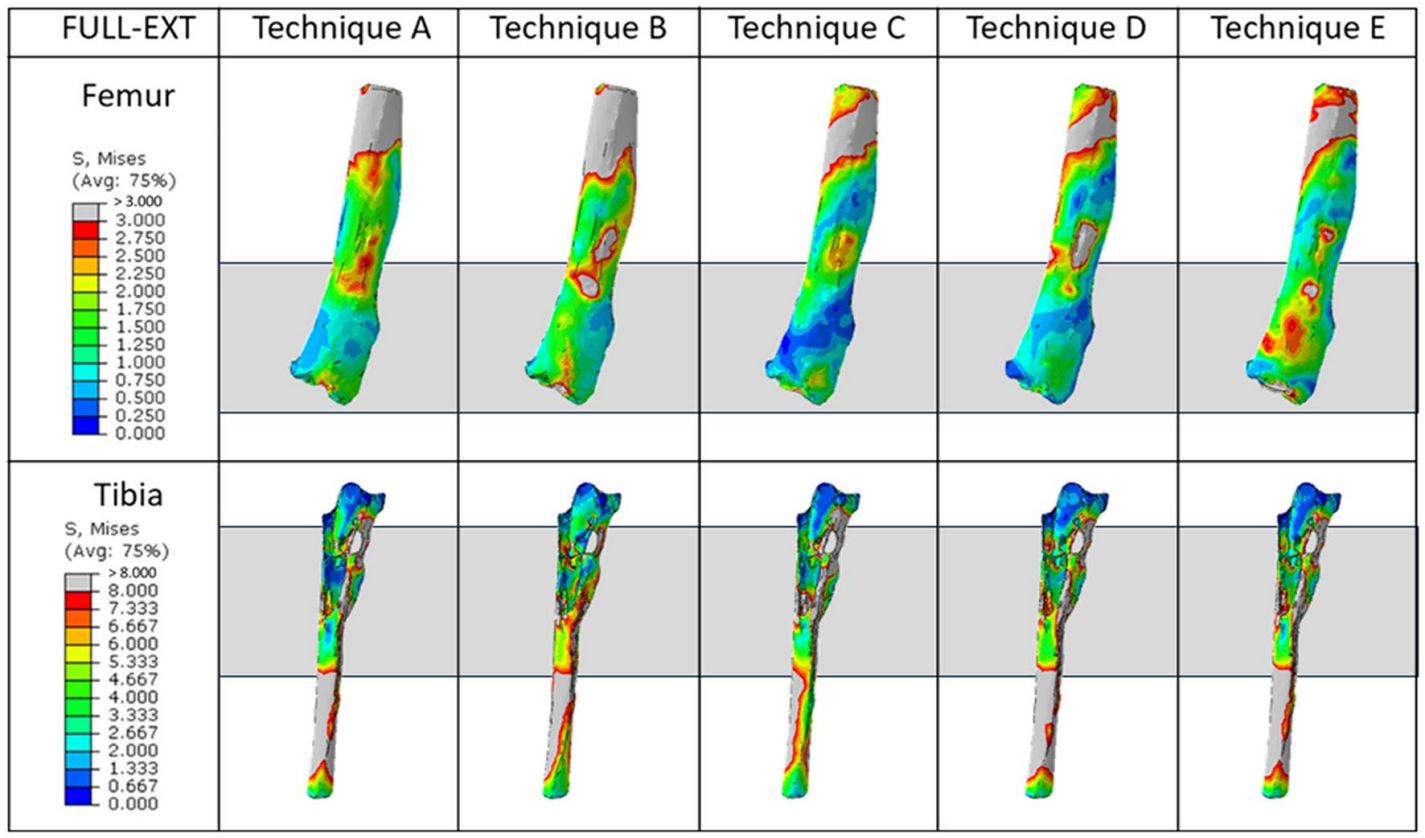
Graphical overview of the von Mises stress for the full-extension configuration. Each column represents the different techniques analyzed, while the rows indicate the different bones. The dark grey rectangles highlight the region of interest considered

Figure 7 shows the qualitative trends for stress in the patient’s remaining bones in the full-extension configuration. Observing the femur in the full-extension configuration, techniques A and B are able to uniformly distribute the stresses, generating a homogeneous stress contour on both bones; on the opposite, the other three techniques tend to concentrate the stress in local areas, such as the stem-bone interface (for techniques C and D) or the extension element-bone interface (for technique E).

**Fig. 8:**
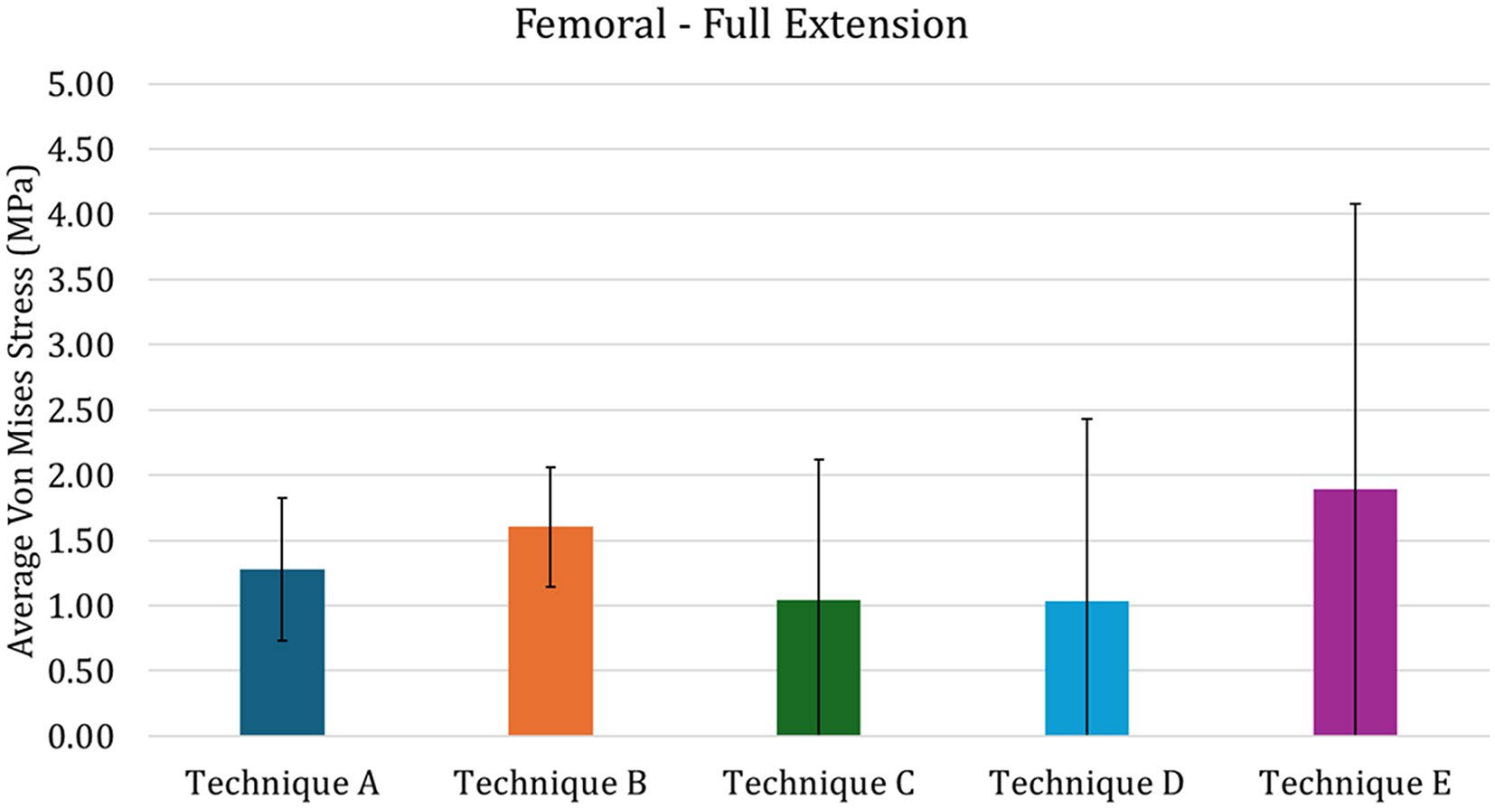
Average von Mises stress in the Distal Femur for the full-extension configuration. The vertical bars represented the standard deviation

**Fig. 9:**
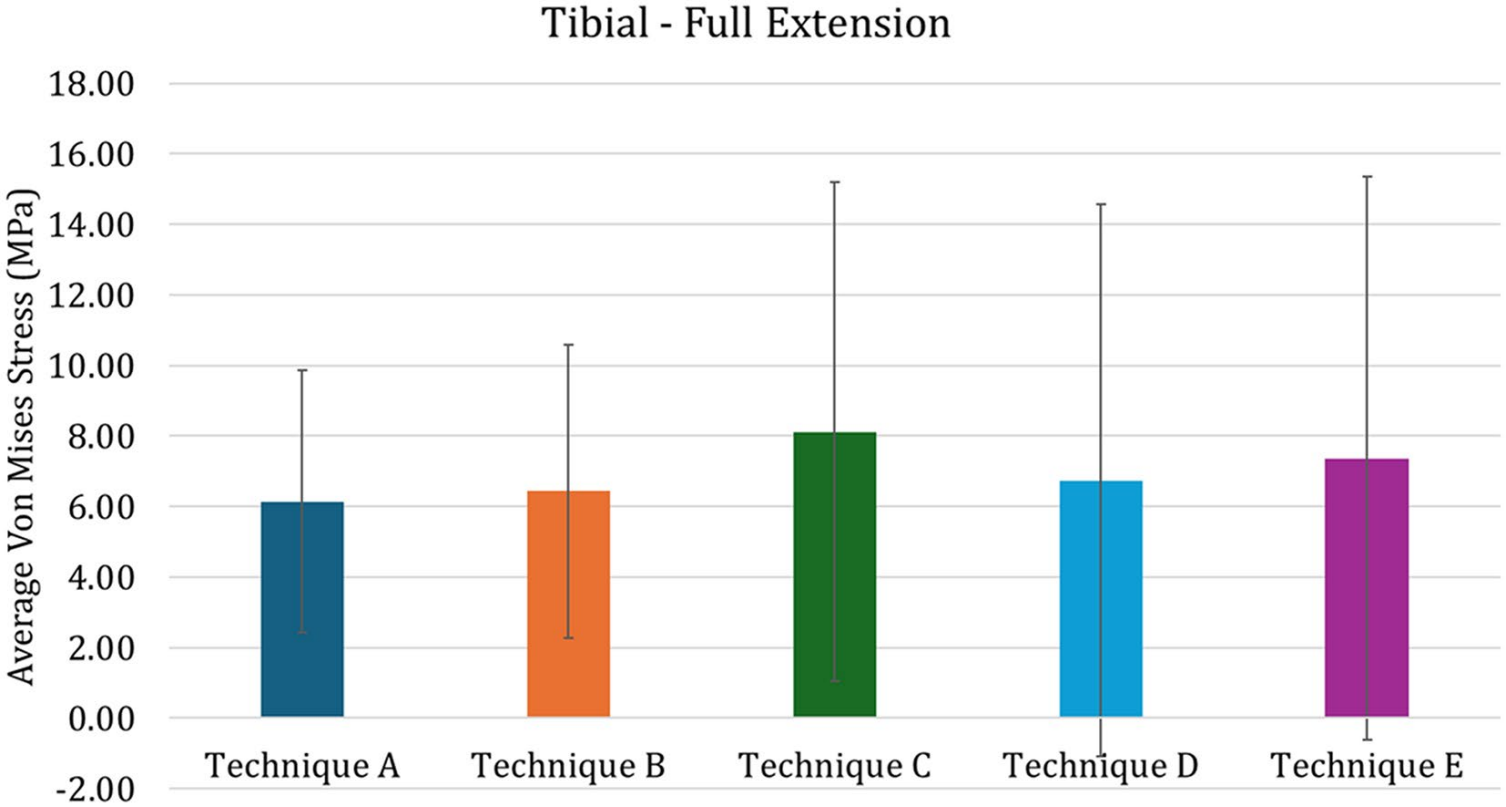
Average von Mises stress in the proximal Tibia for the full-extension configuration. The vertical bars represented the standard deviation

If the use of the custom-made cone is able to produce an uniform stress distribution in the distal femur, on the other hand the presence of the stem tends to induce stress shielding, especially in case of press-fit approach. This can be evaluated in Fig. 8, that report the average Von Mises Stress in the distal femoral region of interest, illustrated in Fig. 7. In the figure it is possible to see how the Techniques C and D are characterized by lower average stresses, while Technique E is characterized by higher values. Comparing techniques A and B, it is possible to highlight a slightly higher average stress obtained in the Technique B: this is mainly due to the change in stiffness which helps in reducing the stress shielding effect. Addressing the variability of the stress, technique E is characterized by higher values, meaning that the max strass values are also higher. Quantitatively addressing the proximal tibia (Fig. 9), the press-fit stem (Technique C) induces higher stress compared to the other techniques. Both cone models (Techniques A and B), regardless of the change in material porosity, exhibit behavior similar to the cemented stem (Technique D).

These findings may occur as the vertical load applied to the bone is mainly transferred to the implant, factor intrinsically due to the device design itself the bone, receiving minimal load, could therefore undergo degeneration (leading to femoral bone resorption).

Furthermore, the use of large resection prostheses coupled with press-fit stem induces stress, but since the distribution is discontinuous, this can lead to local stress peaks that could result in bone weakening or even bone fracture.

**Fig. 10:**
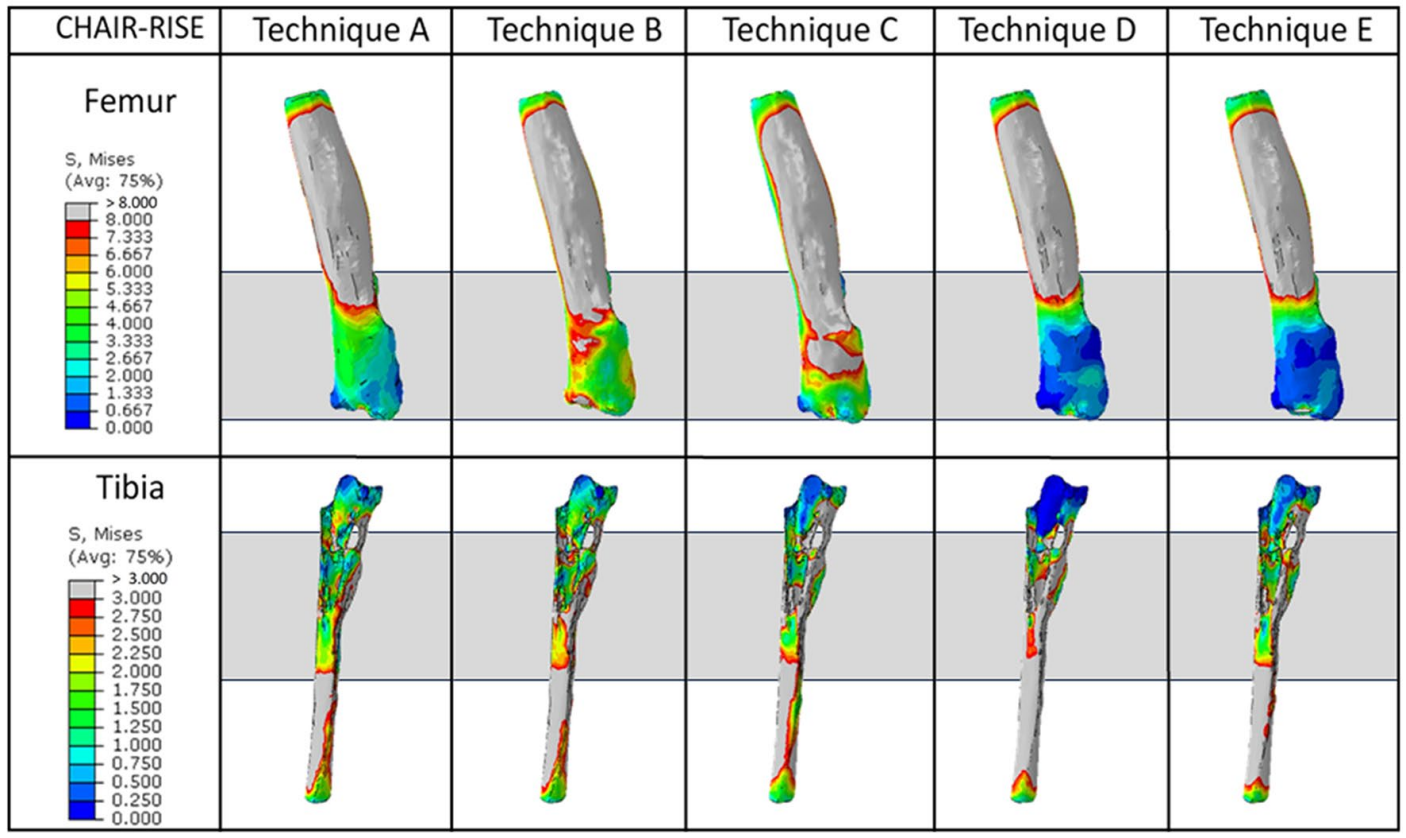
Graphical overview of the von Mises stress for the full-extension configuration. Each column represents the different techniques analyzed, while the rows indicate the different bones. The dark grey rectangles highlight the region of interest considered

Figure 10 illustrates the qualitative trends for stress in the patient’s remaining bones in the chair-rise configuration.

As observed for the full-extension, also for this other activity the use of a custom-made cone induces a more homogeneous stress distribution.

In detail, comparing techniques A and B, the stress induced in the distal femur appears to be uniform in both cases, with a higher stress level in case of porous custom-made cones (Technique B) than in case of conventional ones (Technique A). The technique involving press-fit stem generated high stress concentration that could be a hazard for the increased risk of fracture. Instead, the use of cemented stem and of the large resection prosthesis induces stress shielding in the distal femur.

**Fig. 11:**
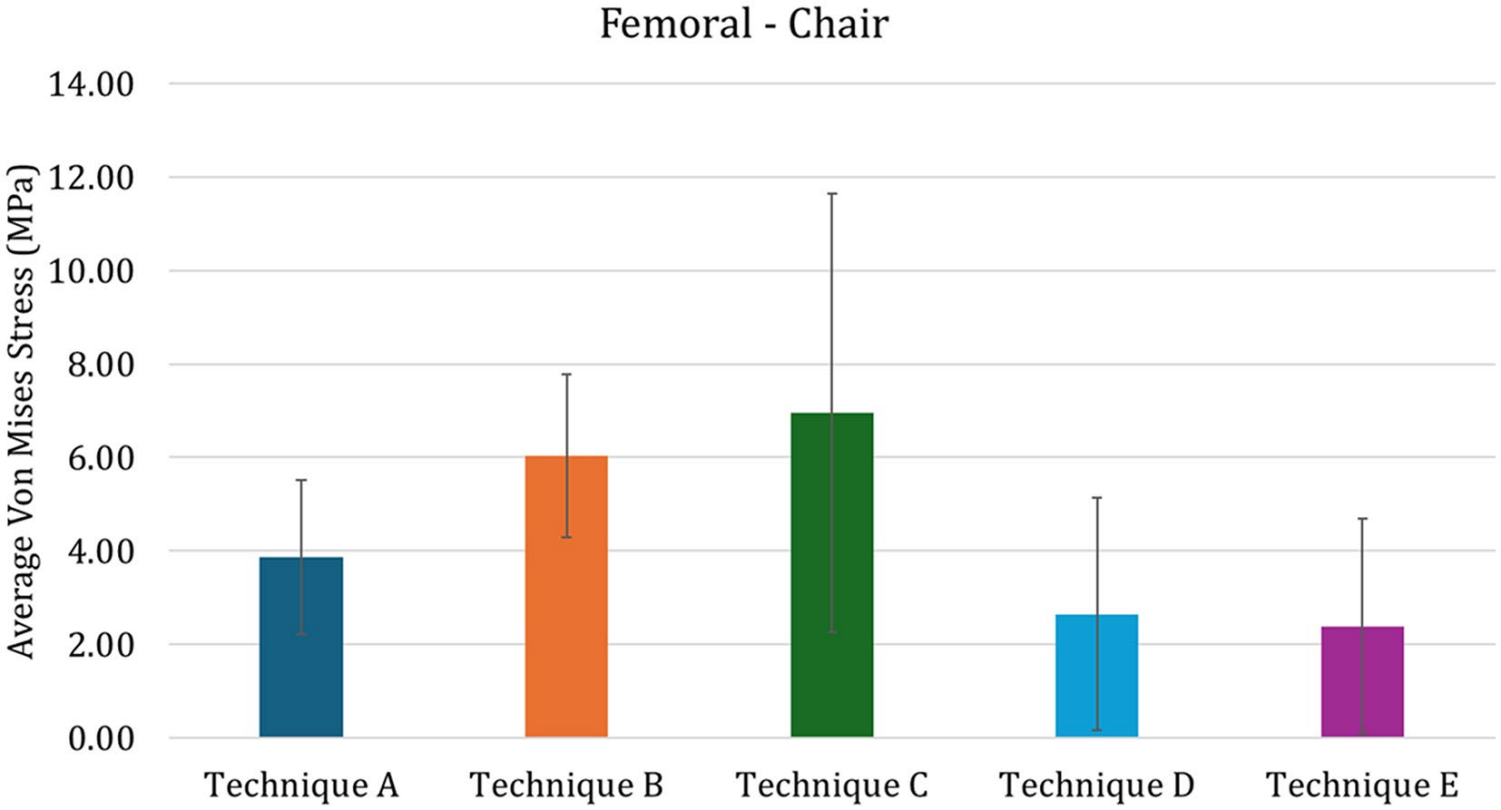
Average von Mises stress in the proximal femur for the Chair configuration. The vertical bars represented the standard deviation

**Fig. 12:**
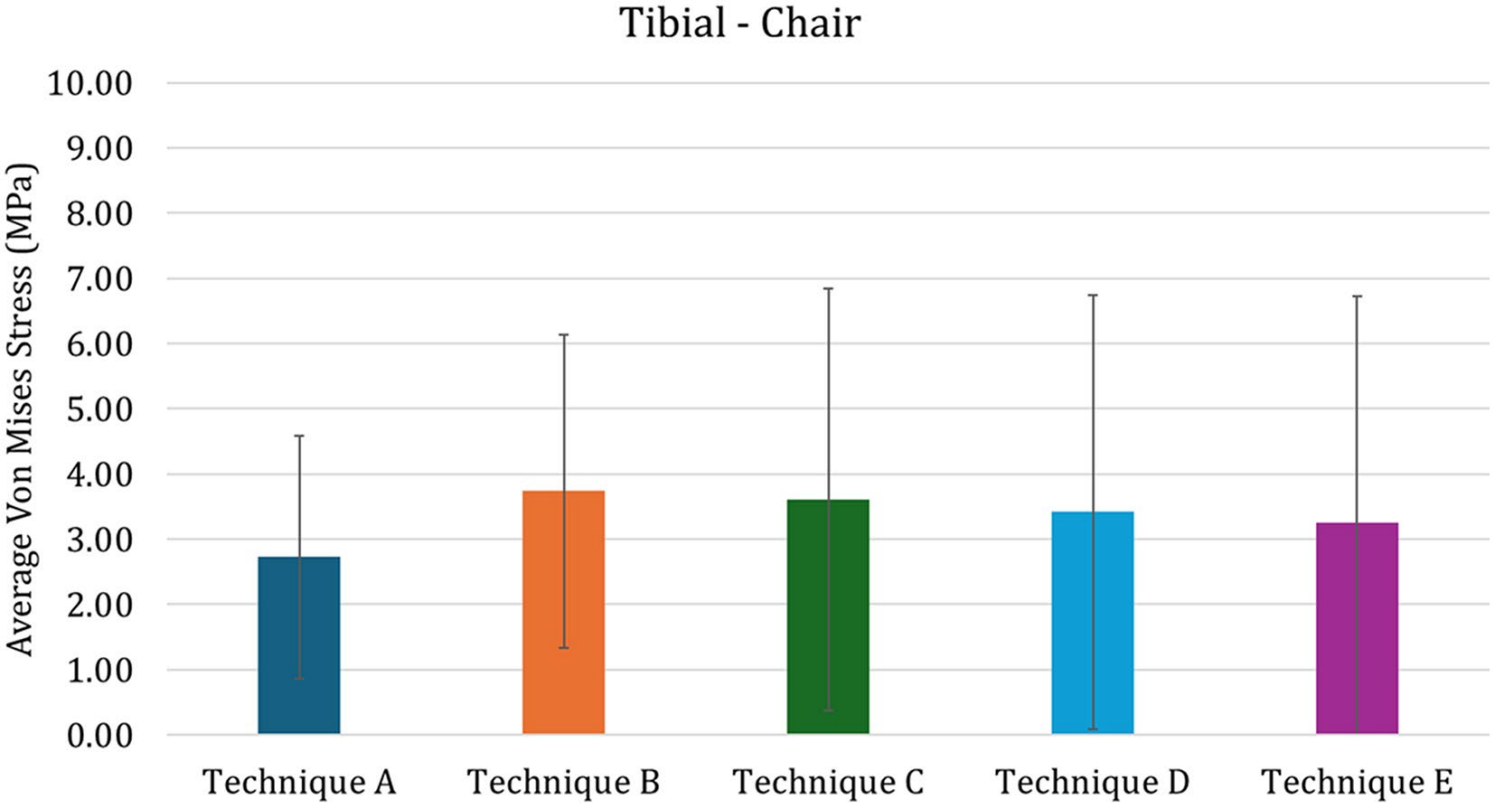
Average von Mises in the distal tibia stress for the Chair configuration. The vertical bars represented the standard deviation

These results could be also quantitatively highlighted in Fig. 11, in which the average Von Mises Stress in the distal femoral region of interest are reported. In this figure it is possible to see that the Technique D and E are characterized by lower average stress, while Technique C is characterized by higher values. Assessing the variability of the stress, technique C is also characterized by higher values, meaning that the max strass values are also higher, while the other values of variability are comparable among techniques. Addressing quantitatively the tibia (Fig. 12), the results are similar to the ones found for the full-extension (Fig. 9): this can be recognized as logical, since the mechanical conditions are almost identical apart for the lower value of force (explaining the overall lower values for average stress).

**Fig. 13:**
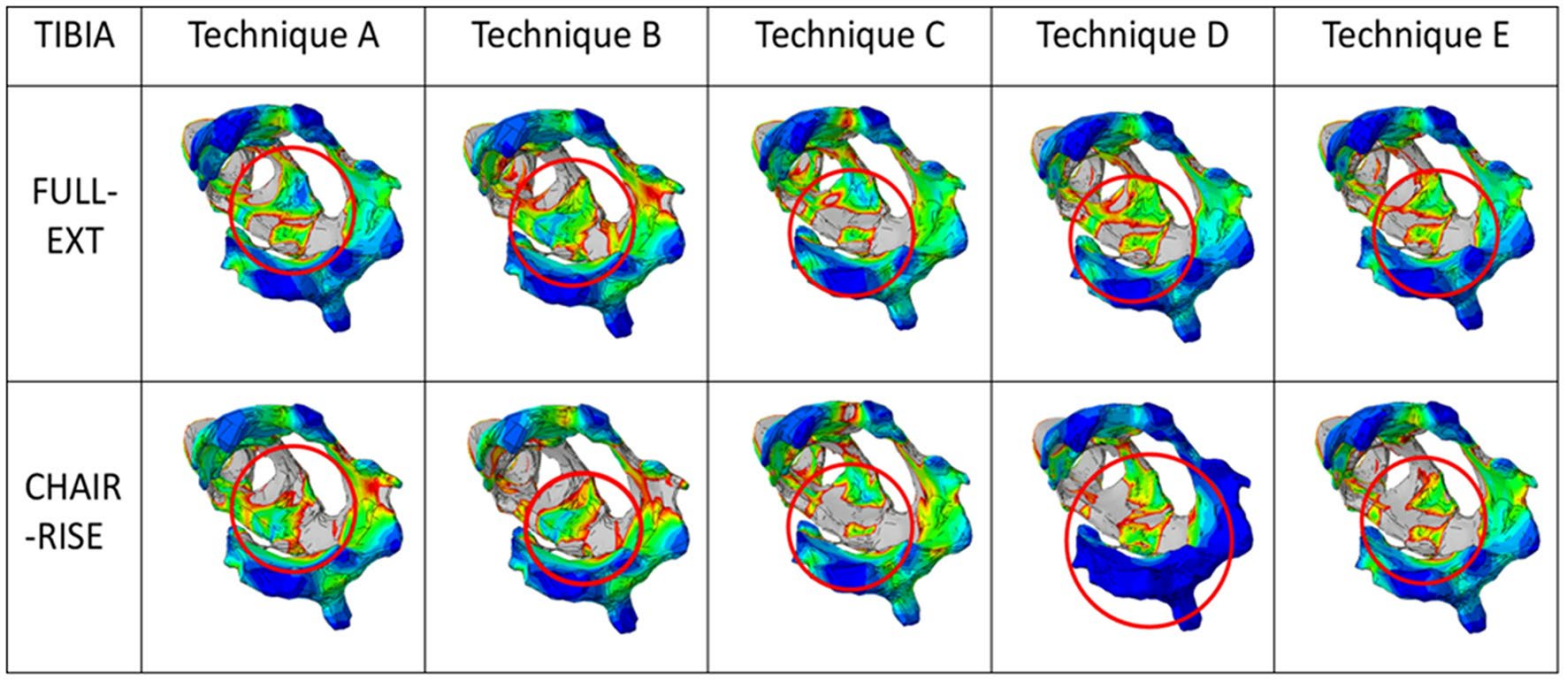
Graphical overview of the von Mises stress in the proximal tibia for the full-extension and chair-rise configuration. Each column represents the different techniques analyzed, while the rows indicate the different tasks. The red circles highlight the region of interest considered

As far as the tibia is concerned, in both full extension and chair-rise configurations (Fig. 13), a better distribution is observed for the revision techniques with custom-made cones rather than in traditional ones, for which the proximal areas of the tibia are under-stressed and may lead to stress-shielding issues.

**Fig. 14:**
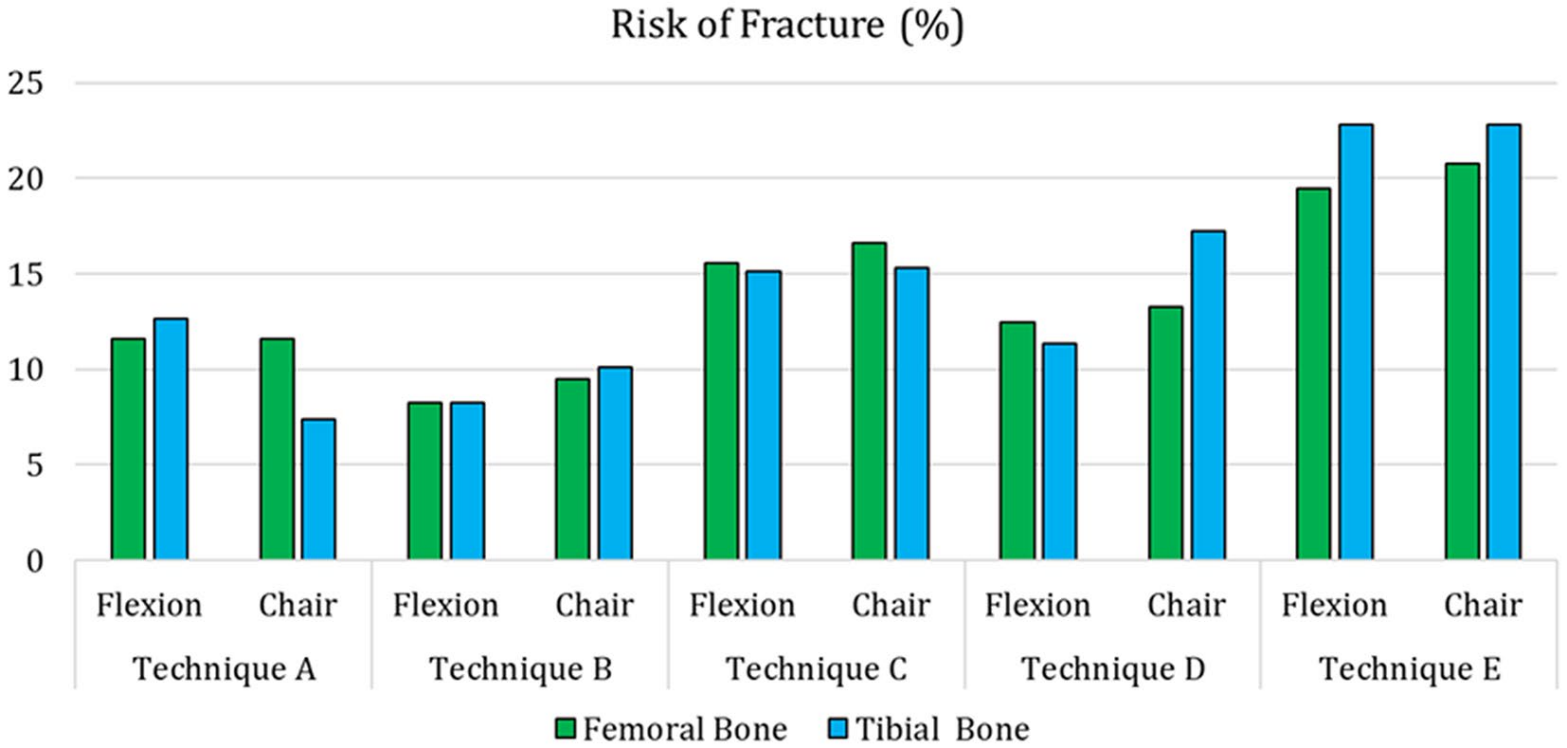
Risk for fracture estimated for the different techniques. The different colors represent the different boundary conditions investigated

Figure 14 report the estimated risk of fracture for the four different techniques investigated in the study. From the graph, it is possible to observe that Techniques A and B generally exhibit lower values of RF, with Technique B being the lowest, and thus safer. Techniques C and D presented slightly higher values of the risk of fracture, with Technique D (cemented stem) being slight lower compared to C (press-fit stem). Technique E is characterized by higher values, although all values are qui lower, with each being far below 100%, as the analyzed motor tasks are based on daily activities usually not dangerous for the patient.

## Discussion

In the revision surgery, prosthetic stability is crucial. Large bone defects and reduced bone availability can make reconstruction and fixation difficult. The management of significant bone tissue loss is therefore one of the primary challenges in knee revision surgery, and custom-made cones represent a new and promising option in this surgical context [14, 15].

All investigated surgical techniques are currently utilized in the clinical field of the R-TKA, with a variety of results. Anyway, extremely complex patient conditions tend to require markedly patient-specific approaches in order to achieve successful results.

Our biomechanical analysis reported favorable outcomes for a patient with major distal femoral and proximal tibial bone losses, whose magnitude was so severe that it was not recommended employing two or more standard cones or resorting to a conventional revision procedure for implant stabilization.

The custom-made cones are designed using the 3D reconstruction of the anatomy deduced from the patient’s tomographic images, thus becoming adaptable for any different bone morphology.

Besides, the combination of tailored geometries with the possibility of modifying the material mechanical properties to suit the patient’s needs represents an excellent opportunity.

These statements are thus supported by the results obtained from this biomechanical analysis: the bone stress observed with the use of custom-made cones is more homogeneously distributed than stress induced by the other traditional techniques, which tend to present stress concentration in specific regions and under-stressed areas. Compared to conventional porosity cones, bi-material cones then returned to be able to better transmit the stress to the surrounding bones then further mitigating the stress-shielding effects that may occur with a prosthesis implant, and reducing the potential risk of fracture.

Morgan-Jones et al. [53] conducted a study on strategies to achieve good fixation of R-TKAs. The authors state that there can be three main types of fixation for knee bones (in the epiphyseal, diaphyseal and metaphyseal zones), and in order to achieve good prosthetic stability, it is necessary to fix more than one zone; this statement finds agreement in other studies in the literature [54]. In this study, the custom-made cones are able to assess the meta-diaphyseal region, i.e. involving both the diaphyseal and metaphyseal canal, therefore providing a reliable prosthetic stability.

These findings are moreover supported by Burastero et al. [15]: the authors reported excellent clinical results in 11 patients, with a follow-up of approximately two years. The custom-made cones appear to be osteointegrated, and radiological images do not show evidence of implant loosening and component migration; other agreeing results can be found in the literature [55].

Lachiewicz et al. [56] published a study on severe bone loss, highlighting the significance of trabecular metal for its beneficial properties, such as a structure that mimics cancellous bone and its superior biocompatibility and osteoconductivity.

Faizar et al. [57] performed then a biomechanical analysis of porous titanium cones, comparing them with cones made of porous tantalum: also in this case, the authors reported results showing greater stability with porous titanium cones. Similarly, Innocenti [37] analyze biomechanically porous cone, and the findings demonstrate that metaphyseal flexible cones are safer in comparison with rigid cones.

The biomechanical results obtained in the present study therefore find and provide support to the positive post-operative clinical outcomes observed in literature for patients who underwent the surgery [14, 15]; in both the techniques involving custom-made cones, indeed, the stress distribution appears to be uniform, reducing local stress peaks that increase friction between the joint surfaces and cause wear of the implant surfaces or even loosening of the prosthesis and reducing the potential risk of fracture. The use of these custom-made cones in revision implants showed thus reduced risks of stress shielding, being able to properly transfer loads to the bone, and increase overall implant stability. As mentioned, these findings can be justified by the mechanical behavior of the cones, which result more similar to the bone mechanical properties and therefore generates a smoother gradient in stress distribution. Additionally, the customized geometry of the prosthesis adapts more effectively to the patient’s anatomy if compared to an off-the-shelves implant geometry.

Comparing the bone stress patterns in the configurations with cones, it is possible to observe the advantages of cones made of high-level porous metal over those made of conventional porous metal. The distal region of the femur and the proximal region of the tibia appear indeed to be less affected by stress shielding in the model involving the high-porous cones rather than the conventional, and this factor can be directly correlated with better osseointegration in the interface area between the bone and the implant.

The findings of the present study support the positive post-operative clinical outcomes observed in the literature for patients who received porous metaphyseal cone [39–41]. specifically, Rossi et al. [39] demonstrated, in a 3 to 11 years follow-up report on 101 patients who underwent 139 metaphyseal cone procedures (80 tibial, 59 femoral). This study reported promising clinical and radiographic results and a high survival rate at mid-term follow up. Hadley et al. [40] in a single-center retrospective study with a 10-year follow up, demonstrated persistently durable longer-term survivorship with a low rate of implant removal when using porous metaphyseal cones. Kayani et al. [41], analyzing 152 patients treated with porous metaphyseal cones, found excellent survivorship, improvements in functional outcomes, and reproducible radiographic osseointegration at mean 5-year follow-up.

It is however worth of mention that this study presents some limitations, mainly related to the fact that only a static analysis was conducted; however, the two simulated load configurations correspond to the most demanding daily activities that a patient with this device and these bone conditions is able to perform, so we can consider these configurations as representative for the sake of comparison.

Moreover, most of the material models are assumed to be linearly elastic: this significant assumption however enables a good approximation of all the mechanical properties, facilitating a qualitative comparison between different configurations, and therefore is typically used for this kind of analyses in the literature [15, 17, 18, 29, 37, 43, 44].

The Risk of Fracture was determined based on the values of the local maximum strain and could therefore be influenced by the mesh used and to the potential element distortion in the contact region. To mitigate this effect as much as possible, a meticulous selection of the mesh of the contact region was conducted, and the proper meshing quality was ensured through convergence analysis [50].

Furthermore, soft tissue and the patella bone were not explicitly included in the model; however, their biomechanical contributions were kept in account as their relative effects were incorporated in the definition of the loads and the boundary conditions, and can therefore be considered as a part of the implemented model.

Finally, it is however to be kept into account that the study’s aim is to achieve a comparative analysis between the different models: all assumptions and approximations mentioned have indeed been equally applied to all models considered, and therefore this approach ensures that any limitations of the model affect each one of the analyzed configurations in the same way, thus minimizing the impact of such limitations on the relative comparison of results.

In summary, the validity of the results is based on a previously validated numerical model [18, 42] and the consistent application of the same assumptions across all compared models. This allows the results obtained to be considered as robust and reliable within the scope of the assumptions made.

In this study, an actual patient was selected to address a genuine clinical situation; while the choice of using a single patient could be seen as a limitation, the fact that the specific patient addressed is characterized by a particularly severe condition allowed for the exploration of a worst-case scenario. For this reason, despite it is to be acknowledged that including multiple patients (with different geometries and varying degrees of severity) could provide additional insights to the literature, the relevance of the present study is therefore not significantly impacted by this limitation. This analysis is therefore able to provide interesting comparative insights and could indeed serve as a starting point for future research involving a broader range of patient cases and conditions.

## Conclusions

In conclusion, the study provides insights into how different custom designs perform under various conditions, allowing clinicians to make informed decisions about the most appropriate design for a specific patient scenario. By understanding the comparative performance of each design, it becomes possible to tailor the surgical approach to better suit the unique anatomical and biomechanical requirements of individual patients. This study succeeded therefore in the aim of providing such support to clinicians in finding the proper surgical solution for those patients with severe bone degeneration, and for which the main solution used to be amputation [58]. Being able to rely on this new kind of implants, capable of adapting to the patient’s bone morphology, replacing bone defects, and promoting osseointegration through the choice of appropriate materials, represent therefore a great advantage for the clinicians and the knowledge of their contribution to the patient biomechanics should be kept into account when defining the optimal tailored treatment. The use of custom-made cones can thus be considered a viable option to manage the patient’s bone loss, and even more so if the structure of the devices is characterized by a higher level of porosity in the region in contact with the bone.

## Data Availability

The data that support the findings of this study are available from the corresponding author, [BI], upon reasonable request.
